# A Revised Structure and Assigned Absolute Configuration of Theissenolactone A

**DOI:** 10.3390/molecules25204823

**Published:** 2020-10-20

**Authors:** Melissa M. Cadelis, Soeren Geese, Lauren Gris, Bevan S. Weir, Brent R. Copp, Siouxsie Wiles

**Affiliations:** 1 School of Chemical Sciences, University of Auckland, Private Bag 92019, Auckland 1142, New Zealand; b.copp@auckland.ac.nz; 2 Bioluminescent Superbugs Lab, School of Medical Sciences, University of Auckland, Private Bag 92019, Auckland 1142, New Zealand; s.geese@auckland.ac.nz; 3 Department of Fine and Industrial Organic Chemistry, SIGMA Clermont, Campus des Cézeaux, CS 20265, 63178 Aubière, France; lauren.gris85@laposte.net; 4 Manaaki Whenua-Landcare Research, Private Bag 92170, Auckland 1142, New Zealand; WeirB@landcareresearch.co.nz

**Keywords:** fungi, antimicrobial, lactone, natural product

## Abstract

Antimicrobial bioassay-guided fractionation of *Microcera larvarum* led to the isolation of a γ-lactone with a furo[3,4-*b*]pyran-5-one bicyclic ring system (**1**) and three known compounds, (3*S*,4*R*)-4-hydroxymellein (**2**), (3*S*,4*S*)-4-hydroxymellein (**3**) and 7-hydroxy-3-(1-hydroxyethyl)isobenzofuran-1(3*H*)-one (**4**). Structure elucidation was conducted by NMR spectroscopic methods. Absolute configuration of **1** (2*R*, 3*S*, 5*S*, 7*S,* 8*R*) was established using the chiral derivatizing agent MPA and was fully supported by calculated specific rotation and ECD spectra. The spectroscopic data observed for **1** were identical to those previously reported for theissenolactone A (**7**), necessitating a correction of the latter (from C-5/C-8 *trans* ring fusion to *cis*). Compounds **1**–**4** were evaluated for antimicrobial activity against a panel of pathogens.

## 1. Introduction

**Figure 1:**
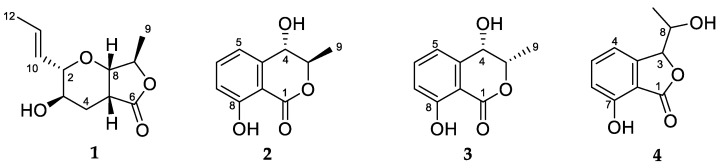
Structures of natural products **1**–**4**.

Most antibiotics used in the clinic today come from soil microbes, beginning with the discovery of penicillin from the fungus *Penicillium*. Aotearoa New Zealand has a treasure trove of unique fungi that have not been exhaustively searched for new antibiotics. Thus, we have recently begun investigating fungal isolates from the International Collection of Microorganisms from Plants (ICMP), which contains cultures derived from plants and soil from Aotearoa New Zealand and the South Pacific, in pursuit of novel antimicrobials. Bioassay-guided investigation of the ascomycete fungus *Microcera larvarum* led to the isolation of a γ-lactone with a furo[3,4-*b*]pyran-5-one bicyclic ring system (**1**) and three other known natural products including two hydroxymelleins (**2** and **3**) and an isobenzofuranone (**4**) (Figure 1).

**Figure 2:**
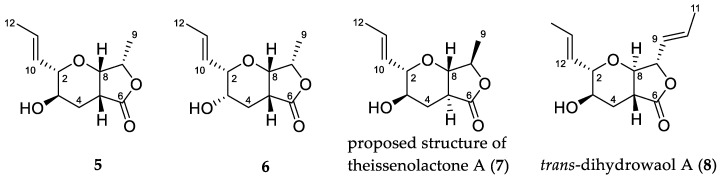
Structures of reported γ-lactones **5**–**8**.

Literature search of γ-lactones with the same furo[3,4-*b*]pyran-5-one bicyclic ring system as **1** identified three isomeric compounds, **5**–**7** (Figure 2), previously reported from fungi. The planar structure of **1** was first reported by Gao et al. as synthetic intermediates **5** and **6** in their study of a related natural product, TAN-2483A [1]. A third example of this planar structure is that of theissenolactone A (**7**), reported by Liang et al. from the fermentation broth of *Theissenia cinerea* 89,091,602 [2]. Of note in the case of **7** is the reported C-5/C-8 trans ring fusion. The relative configuration of **5**–**7** have been reported while the absolute configuration has yet to be established.

Structure verification of natural products **1**–**4** was established by spectroscopic methods while the absolute configuration of **1** was assigned by use of a chiral derivatizing agent and further supported by computational chemistry. Direct comparison of the spectroscopic data of **1** and **7** [2] showed that they were identical. Herein, we report the revised structure of theissenolactone A from **7** to **1** and establishment of the absolute configuration of this compound.

## 2. Results and Discussion

Freeze-dried agar plates inoculated with *Microcera larvarum* were extracted using a combination of methanol and dichloromethane. Initial fractionation of the crude organic extract was conducted by C_8_ reversed-phased column chromatography, eluting with gradient H_2_O/MeOH which afforded five fractions (F1–F5). Antimicrobial testing of F1–F5 against *Staphylococcus aureus* ATCC 29,213 and *Escherichia coli* ATCC 25,922 identified activity for F3 and F4 against the latter at minimum inhibitory concentrations (MIC) of 1 and 0.5 mg/mL, respectively. In addition, F4 also exhibited non-bactericidal activity against *S. aureus* at MIC 1 mg/mL. Further purification of F2–F4 by a combination of Diol-bonded silica and silica gel column chromatography led to the isolation of compounds **1**–**4**.

**Figure 3:**
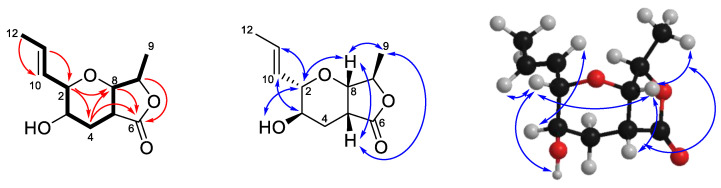
Selected COSY (bold), HMBC (**red arrows**) and NOESY (**blue arrows**) correlations for **1**.

Compound **1** was isolated as a white solid. High resolution ESI mass spectrometry identified a sodiated adduct of *m/z* 235.0940 [M + Na]^+^ which was consistent with the molecular formula C_11_H_16_O_4_ (Figure S10). Analysis of the ^1^H-NMR spectrum in CDCl_3_ (Table 1, Figure S4) identified the presence of two *trans* olefinic protons (δ_H_ 5.89 (dq, *J* = 15.5, 6.6 Hz, H-11) and 5.42 (ddq, *J* = 15.5, 7.7, 1.6 Hz, H-10)), four oxymethines (δ_H_ 4.58 (q, *J* = 7.0 Hz, H-7), 4.04 (d, *J* = 4.1 Hz, H-8), 3.45 (dd, *J* = 9.0, 7.7 Hz, H-2) and 3.34 (ddd, *J* = 11.0, 9.0, 5.0 Hz, H-3)), a methine (δ_H_ 2.90 (ddd, *J* = 6.1, 4.1, 2.0 Hz, H-5)), a diastereotopic methylene (δ_H_ 2.64 (ddd, *J* = 13.4, 5.0, 2.0 Hz, H_2_-4_A_) and 1.73 (ddd, *J* = 13.4, 11.0, 6.1 Hz, H_2_-4_B_)) and two methyl groups (δ_H_ 1.77 (dd, *J* = 6.6, 1.6 Hz, H_3_-12) and 1.33 (d, *J* = 7.0 Hz, H_3_-9)). The ^13^C-NMR data (Table 2, Figure S5) revealed 11 carbon signals, indicative of one carbonyl (δ_C_ 176.0 (C-6)), two olefinic (δ_C_ 127.8 (C-10) and 133.0 (C-11)), four oxymethine (δ_C_ 66.6 (C-3), 77.7 (C-8), 80.5 (C-7), 81.5 (C-2)), one methine (δ_C_ 39.0 (C-5)), one methylene (δ_C_ 28.3 (C-4)) and two methyl carbons (δ_C_ 17.6 (C-9) and 18.2 (C-12)). The COSY spectrum established the connectivity order as H_3_-12 to H-11 to H-10 to H-2 to H-3 to H_2_-4 to H-5 to H-8 to H-7 to H_3_-9 (Figure 3). Key HMBC correlations were observed between H-7 and C-6, H-8 and C-6, and H_2_-4 and C-6, as well as a weak correlation between H-2 and C-8, which showed connectivity along the fused lactone-furanone fragment. Thus, the planar structure of **1** was established as shown. Establishment of the relative configuration of **1** was conducted using NOESY correlations, which were observed from H_3_-9 to H-5 to H-8 to H-2 to OH, indicating that these protons were on the same face of the compound. An additional correlation from H-3 to H-10 indicated that these two protons were on the same face to each other but on the opposite face to the previous set establishing the relative configuration of **1** as H_3_-9/H-5/H-8/H-2/OH as α orientation while H-3/H-7/H-10 as β orientation.

Since it was noted that **1** and **5**–**7** were isomeric, the spectroscopic data of these compounds were compared. Both the ^1^H and ^13^C-NMR data of **1** (Table 1 and Table 2) were very similar to those of synthetic intermediate **5** with the exception of H_3_-9. For **1**, H_3_-9 was observed at δ_H_ 1.33 with a carbon signal of δ_C_ 17.6, while, for **5**, the proton signal was observed at δ_H_ 1.44 and the corresponding carbon at δ_C_ 13.5. This locus of difference suggested that **1** is the C-7 epimer of **5**. The NMR data of compound **6** showed significant changes in comparison to **1** around both the lactone and furanone rings as expected since **6** is epimeric with **1** at C-3 and C-7.

Comparison of the ^1^H and ^13^C-NMR data of **1** in CD_3_OD (Table 3, Figures S2 and S3) with theissenolactone A (**7**) showed that the data were identical [2]. This was surprising as the reported relative configuration of **7** indicated that **1** should be the *cis* isomer of **7** at the H-5/H-8 bridge. Examination of the coupling constants across the H-5/H-8 bridge showed that for **7** H-8 was observed as a singlet [2]. This was inconsistent with previously reported data for *trans* bridge head protons, for a related natural product *trans*-dihydrowaol A (**8**), which had a coupling constant of *J* = 12 Hz [3]. In the current study, we observed a small coupling constant of *J* = 4.3 Hz for the H-5/H-8 bridge which was consistent with the coupling constants previously published for *cis* isomers **5** *(J* = 3.1 Hz) and **6** (*J* = 3.6 Hz) [1]. The optical rotation of **7**, [α]_D_^28^ = +47.7 (*c* = 0.45, MeOH) [2], was higher in magnitude in comparison to **1**, [α]_D_^22^ = +23.7 (*c* = 0.16, MeOH), but was of the same sign. Taken together, these results indicate **1** and **7** are the same compound and thus the correct structure of theissenolactone A is **1**.

**Figure 4:**
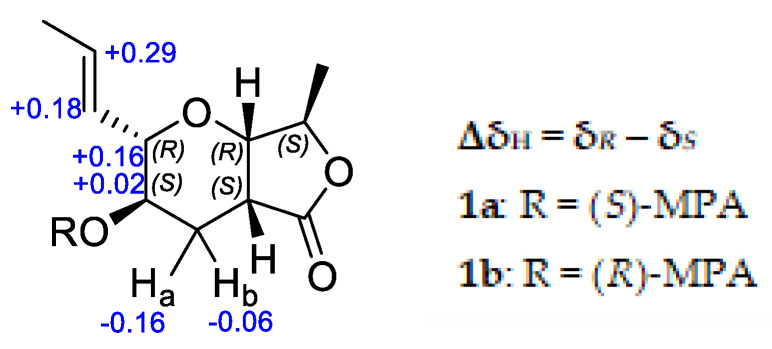
Δδ_H_ values obtained for (*S*)-MPA and (*R*)-MPA ester derivatives (**1a** and **1b**, respectively) of **1** in CDCl_3_.

**Figure 5:**
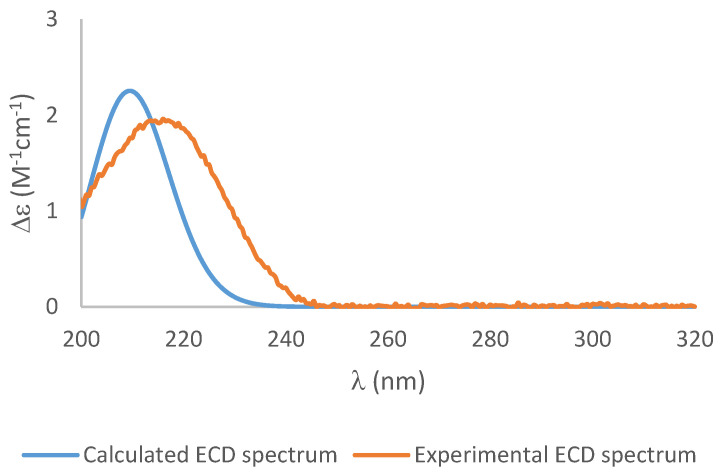
The experimental ECD spectrum (**orange**), and the calculated ECD spectrum of **1** (**blue**) (blue shift = −6).

To establish the absolute configuration of **1**, the chiral derivatizing agent α-methoxyphenylacetic acid (MPA) was used to prepare diastereomeric esters at C-3. Treatment of **1** with (*S*)-MPA and (*R*)-MPA, respectively, in the presence of EDC.HCl and DMAP afforded derivatives **1a** and **1b**. Comparison of the ^1^H-NMR spectra of the two compounds (Figure 4), especially those of H_2_-4, H-3, H-2, H-10 and H-11, identified a change in δ*(R)-*δ*(S)* as positive, establishing *S* configuration at H-3 [4]. Thus, the absolute configuration of **1** was assigned as 2*R*, 3*S*, 5*S*, 7*S,* 8*R*. Further confirmation of the absolute configuration of **1** was derived from computational chemistry. Boltzmann-population weighted (Table S2) specific rotation calculated at the CAM-B3LYP/aug-cc-PVTZ level of theory agreed in sign and magnitude (+45.8) with the reported values for **1** (+47.7 [2], +23.7 (current study)). The electronic circular dichroism spectrum of **1** was acquired as exhibited a single absorbance at λ_max_ 217 nm (Δε 1.94) (Figure 5). Using the same Boltzmann-population weighting, the TDDFT calculated ECD spectrum, calculated at the CAM-B3LYP/def2SVP level of theory, was in close agreement (Δε 2.25, λ_max_ 210 nm) with the observed spectrum.

The structures of the known compounds were identified as (3*S*,4*S*)-4,8-dihydroxy-3-methylisochroman-1-one (**2**) [5], (3*S*,4*R*)-4,8-dihydroxy-3-methylisochroman-1-one (**3**) [5] and 7-hydroxy-3-(1-hydroxyethyl)isobenzofuran-1(3*H*)-one (**4**) [6].

Pure compounds **1**–**3** were tested for antimicrobial activity against a panel of bacteria including Methicillin-resistant *Staphylococcus aureus, Pseudomonas aeruginosa, Escherichia coli, Klebsiella pneumoniae* and *Acinetobacter baumanii* as well as two fungal strains, *Candida albicans* and *Cryptococcus neoformans* (Table 4). None of the compounds exhibited activity at a dose of 32 µg/mL.

## 3. Materials and Methods

### 3.1. General Experimental Procedures

Infrared spectra were recorded on a Perkin-Elmer spectrometer 100 Fourier Transform infrared spectrometer equipped with a universal ATR accessory. HRMS data were acquired on a Bruker micrOTOF QII spectrometer. NMR spectra were recorded on a Bruker Avance DRX-400 spectrometer or an Avance III-HD 500 spectrometer operating at 400 or 500 MHz for ^1^H nuclei and 100 or 125 MHz for ^13^C nuclei. Proto-deutero solvent signals were used as internal references (DMSO-*d*_6_: δ_H_ 2.50, δ_C_ 39.52, CD_3_OD: δ_H_ 3.31, δ_C_ 49.00 and CDCl_3_: δ_H_ 7.26, δ_C_ 77.16). For ^1^H NMR, the data are quoted as position (δ), relative integral, multiplicity (s = singlet, d = doublet, t = triplet, q = quartet, m = multiplet, br = broad), coupling constant (*J*, Hz) and assignment to the atom. The ^13^C-NMR data are quoted as position (δ) and assignment to the atom. Flash column chromatography was carried out using either Merck Diol bonded silica (40–63 µm), Davisil silica gel (40–63 µm) or Merck C_8_ reversed-phase (40–63 µm) solid support. Thin layer chromatography was conducted on Merck DC-plastikfolien Kieselgel 60 F254 plates. All solvents used were of analytical grade or better and/or purified according to standard procedures.

### 3.2. Fungal Material

All fungal isolates were provided by Manaaki Whenua—Landcare Research responsible for the curation of the International Collection of Microorganisms from Plants (ICMP). The ascomycete fungus *Microcera larvarum*, culture ICMP 5444, was isolated from a dead scale insect in December 1974 in Tauranga, Aotearoa New Zealand [7]. Fungal isolates were stored individually in cryotubes at −80 °C. Freezer stocks were made by growing the fungus on 1.5% potato dextrose agar (PDA) plate and excising small cubes of agar (5–6 mm in length) from the fungus’ growing edge. These cubes were placed within a cryovial containing 1 mL of 10% glycerol. The cryovials were rested for 1 h after which the remaining liquid glycerol was removed, and the tubes stored at −80 °C.

### 3.3. Fermentation, Extraction and Isolation

Forty PDA plates were inoculated with ICMP 5444 and incubated at room temperature for 4 weeks. Fully grown fungal plates were freeze-dried (13.40 g, dry weight) and extracted with MeOH (2 × 500 mL) for 4 h followed CH_2_Cl_2_ (2 × 500 mL) overnight. Combined organic extracts were concentrated under reduced pressure to afford a yellow oil (0.51 g). The crude product was subjected to C_8_ reversed-phase column chromatography eluting with a gradient of H_2_O/MeOH to afford five fractions (F1–F5). F2 was subjected to purification by Diol-bonded silica gel column chromatography, eluting with gradient *n*-hexane/EtOAc to afford 7-hydroxy-3-(1-hydroxyethyl)isobenzofuran-1(3*H*)-one (**4**) (1.50 mg). Purification of F3 by Diol-bonded silica gel column chromatography, eluting with gradient *n*-hexane/EtOAc, afforded four fractions (A1–A4). Fraction A2 afforded (3*S*,4*S*) 4-hydroxymellein (**2**) (9.0 mg) while fraction A3 afforded (2*R*,3*S*,4a*S*,7*S*,7a*R*)-3-hydroxy-7-methyl-2-((*E*)-prop-1-en-1-yl)hexahydro-5*H*-furo[3,4-*b*]pyran-5-one (**1**) (3.09 mg) after further purification by silica gel column chromatography, eluting with gradient *n*-hexane/EtOAc. Purification of F4 by Diol-bonded silica gel column chromatography, eluting with gradient *n*-hexane/EtOAc, afforded (3*S*,4*R*) 4-hydroxymellein (**3**) (4.0 mg).

#### 3.3.1. (2R,3S,4aS,7S,7aR)-3-Hydroxy-7-methyl-2-((E)-prop-1-en-1-yl)hexahydro-5H-furo[3,4-b]pyran-5-one (**1**)

White solid; [α]_D_^22^ = +23.7 (*c* = 0.16, MeOH); UV (MeOH) λ_max_ [log *ε*] 318.5 (2.30), 224.0 (2.93), 204.5 (3.38); ECD (MeOH) λ_max_ (Δε) 216 (+1.94); m.p. 129–131 °C; R_f_ 0.70 (EtOAc); IR (ATR) v_max_ 2959, 2949, 2917, 1649, 1457, 1377, 666 cm^−1^; Refer to Table 1 and Table 2 for ^1^H and ^13^C-NMR data; (+)-HRESIMS *m*/*z* 235.0940 [M + Na]^+^ (calcd for C_11_H_16_NaO_4_, 235.0941).

#### 3.3.2. (3S,4R)-4-Hydroxymellein (**2**)

White solid; [α]_D_^19^ = +38.2 (*c* = 0.10, MeOH) [lit [α]_D_^20^ = +32 (*c* = 0.1, MeOH) [5]]; m.p. 124–126 °C; ^1^H-NMR (CD_3_OD, 400 MHz) 7.55 (1H, dd, *J* = 8.4, 7.4 Hz, H-6), 6.97 (1H, d, *J* = 8.4 Hz, H-5), 6.97 (1H, dd, *J* = 7.4 Hz, H-7), 4.72 (1H, qd, *J* = 6.6, 2.0 Hz, H-3), 4.56 (1H, d, *J* = 2.0 Hz, H-4), 1.52 (3H, d, *J* = 6.6 Hz, H_3_-9) [data agreed with literature [5]]; ^1^H-NMR (DMSO-*d*_6_, 400 MHz) δ 10.97 (1H, br s, 8-OH), 7.60 (1H, dd, *J* = 8.5, 8.2 Hz, H-6), 6.99 (1H, dd, *J* = 8.5, 1.0 Hz, H-5), 6.97 (1H, dd, *J* = 8.2, 1.0 Hz, H-7), 5.72 (1H, d, *J* = 6.3 Hz, 4-OH), 4.77 (1H, dq, *J* = 6.6, 2.0 Hz, H-3), 4.52 (1H, dd, *J* = 6.3, 2.0 Hz, H-4), 1.41 (3H, d, *J* = 6.6 Hz, H_3_-9); ^13^C-NMR (DMSO-*d*_6_, 100 MHz) δ 169.0 (C-1), 160.5 (C-8), 142.4 (C-4a), 136.4 (C-6), 118.7 (C-5), 116.7 (C-7), 107.4 (C-8a), 78.3 (C-3), 65.2 (C-4), 15.8 (C-9); (+)-HRESIMS *m*/*z* 217.0476 [M + Na]^+^ (calcd for C_10_H_10_NaO_4_, 217.0470).

#### 3.3.3. (3S,4S)-4-Hydroxymellein (**3**)

White solid; [α]_D_^19^ = +21.2 (*c* = 0.07, MeOH) [lit [α]_D_ = +37.4 (*c* = 0.33, MeOH) [8]]; ^1^H-NMR (CD_3_OD, 400 MHz) δ 7.57 (1H, dd, *J* = 8.5, 7.6 Hz, H-6), 7.08 (1H, d, *J* = 7.6 Hz, H-5), 6.93 (1H, d, *J* = 8.5 Hz, H-7), 4.57–4.53 (2H, m, H-3, H-4), 1.47 (3H, d, *J* = 6.1 Hz, H_3_-9) [data agreed with literature [5]]; ^1^H-NMR (DMSO-*d*_6_, 400 MHz) δ 10.88 (1H, br s, 8-OH), 7.60 (1H, dd, *J* = 8.4, 8.4 Hz, H-6), 7.05 (1H, d, *J* = 8.4 Hz, H-5), 6.94 (1H, d, *J* = 8.4 Hz, H-7), 6.10 (1H, br s, 4-OH), 4.56–4.53 (2H, m, H-3, H-4), 1.39 (3H, d, *J* = 6.0 Hz, H_3_-9); ^13^C-NMR (DMSO-*d*_6_, 100 MHz) δ 169.0 (C-1), 160.5 (C-8), 142.4 (C-4a), 136.4 (C-6), 118.7 (C-5), 116.7 (C-7), 107.4 (C-8a), 78.3 (C-3), 65.2 (C-4), 15.8 (C-9); (+)-HRESIMS *m/z* 217.0475 [M + Na]^+^ (calcd for C_10_H_10_NaO_4_, 217.0470).

#### 3.3.4. 7-Hydroxy-3-(1-hydroxyethyl)isobenzofuran-1(3H)-one (**4**)

White solid; [α]_D_^21^ = −14.7 (*c* = 0.14, CHCl_3_) [lit [α]_D_^20^ = −50.0 (*c* = 0.08, CHCl_3_) [6]]; m.p. 101–103 °C [lit 102–103 °C [6,9]]; ^1^H-NMR (DMSO-*d*_6_, 400 MHz) δ 10.63 (1H, br s, 7-OH), 7.51 (1H, dd, *J* = 8.0, 7.6 Hz, H-5), 7.03 (1H, d, *J* = 7.6 Hz, H-4), 6.90 (1H, d, *J* = 8.0 Hz, H-6), 5.28 (1H, d, *J* = 4.5 Hz, H-3), 5.22 (1H, d, *J* = 5.4 Hz, 8-OH), 3.95–3.91 (2H, m, H_2_-8), 1.05 (3H, d, *J* = 6.5 Hz, H_3_-9); ^13^C-NMR (DMSO-*d*_6_, 100 MHz) δ 168.2 (C-1), 156.8 (C-7), 149.7 (C-3a), 135.7 (C-5), 115.8 (C-6), 113.4 (C-4), 111.7 (C-7a), 83.0 (C-3), 67.5 (C-8), 18.0 (C-9); (+)-HRESIMS *m*/*z* 217.0472 [M + Na]^+^ (calcd for C_10_H_10_NaO_4_, 217.0470).

#### 3.3.5. Preparation of (S)-MPA derivative of **1**

To **1** (0.40 mg, 1.9 µmol) in anhydrous CH_2_Cl_2_ (0.5 mL) under nitrogen atmosphere was added (*S*)-(+)-α-methoxyphenylacetic acid (0.78 mg, 4.7 µmol), EDC.HCl (0.90 mg, 4.7 µmol) and DMAP (0.57 mg, 4.7 µmol) successively and the reaction was stirred overnight. Water was added and the crude product was extracted with diethyl ether (2 × 5 mL). The combined organic extracts were washed with 10% HCl (5 mL), water (5 mL), sat aq. NaHCO_3_ (5 mL) and water (5 mL) successively and dried over anhydrous MgSO_4_. Solvent was then removed under pressure to afford (*S*)-MPA derivative (**1a**) of **1** as a colorless oil (0.40 mg, 58.8% yield). ^1^H-NMR (CDCl_3_, 400 MHz) δ 7.39–7.32 (5H, m, ArH-MPA), 5.37 (1H, dq, *J* = 15.5, 6.6 Hz, H-11), 5.11 (1H, ddq, *J* = 15.5, 7.7, 1.6 Hz, H-10), 4.72 (1H, s, CαH-MPA), 4.69 (1H, ddd, *J* = 11.0, 9.0, 5.0 Hz, H-3), 4.55 (1H, q, *J* = 7.0 Hz, H-7), 3.92 (1H, d, *J* = 4.1 Hz, H-8), 3.51 (1H, dd, *J* = 9.0, 7.7 Hz, H-2), 3.40 (3H, s, OMe-MPA), 2.85 (1H, ddd, *J* = 6.1, 4.1, 2.0 Hz, H-5), 2.54 (1H, ddd, *J* = 13.4, 5.0, 2.0 Hz, H_2_-4_A_), 1.90 (1H, ddd, *J* = 13.4, 11.0, 6.1 Hz, H_2_-4_B_), 1.40 (3H, dd, *J* = 6.6, 1.6 Hz, H_3_-12), 1.29 (3H, d, *J* = 7.0 Hz, H_3_-9); (+)-HRESIMS *m*/*z* 383.1503 [M + Na]^+^ (calcd for C_20_H_24_NaO_6_, 383.1470).

#### 3.3.6. Preparation of (R)-MPA derivative of **1**

To **1** (0.50 mg, 2.4 µmol) in anhydrous CH_2_Cl_2_ (0.5 mL) under nitrogen atmosphere was added (*R*)-(–)-α-methoxyphenylacetic acid (0.98 mg, 5.9 µmol), EDC.HCl (1.13 mg, 5.9 µmol) and DMAP (0.72 mg, 5.9 µmol) successively and the reaction was stirred overnight. Water was added and the crude product was extracted with diethyl ether (2 × 5 mL). The combined organic extracts were washed with 10% HCl (5 mL), water (5 mL), sat aq. NaHCO_3_ (5 mL) and water (5 mL) successively and dried over anhydrous MgSO_4_. Solvent was then removed under pressure to afford (*R*)-MPA derivative (**1b**) of **1** as a colorless oil (0.47 mg, 54.7% yield). ^1^H-NMR (CDCl_3_, 400 MHz) δ 7.41–7.33 (5H, m, ArH-MPA), 5.66 (1H, dq, *J* = 15.5, 6.6 Hz, H-11), 5.29 (1H, ddq, *J* = 15.5. 7.7, 1.6 Hz, H-10), 4.71 (1H, ddd, *J* = 11.0, 9.0, 5.0 Hz, H-3), 4.70 (1H, s, CαH-MPA), 4.57 (1H, q, *J* = 7.0 Hz, H-7), 3.98 (1H, d, *J* = 4.1 Hz, H-8), 3.67 (1H, dd, *J* = 9.0, 7.7 Hz, H-2), 3.40 (3H, s, OMe-MPA), 2.74 (1H, ddd, *J* = 6.1, 4.1, 2.0 Hz, H-5), 2.38 (1H, ddd, *J* = 13.4, 5.0, 2.0 Hz, H_2_-4_A_), 1.84 (1H, ddd, *J* = 13.4, 11.0, 6.1 Hz, H_2_-4_B_), 1.54 (3H, dd, *J* = 6.6, 1.6 Hz, H_3_-12), 1.29 (3H, d, *J* = 7.0 Hz, H_3_-9); (+)-HRESIMS *m*/*z* 383.1468 [M + Na]^+^ (calcd for C_20_H_24_NaO_6_, 383.1470).

### 3.4. Computational Details

Conformational analysis of **1** was performed using the MMFF94 molecular mechanics force field via the Spartan ‘08 program. Seven conformers were identified in a 15 kJ/mol window, which, upon initial geometry optimization at the DFT B3LYP/6-31G(d) level of theory and removal of duplicates, afforded four stable conformers (**1a**–**d**) (Figure S1). Each of these conformers was then re-optimized at the B3LYP/6-311+G(2d,p) level using Grimme’s empirical dispersion corrections [10] with Becke–Johnson damping (D3BJ) [11] with addition of the integral equation formalism variant of Polarizable Continuum Model (IEFPCM, MeOH) in Gaussian 09W [12]. Harmonic vibrational frequencies were calculated for each conformer at the same level of theory to confirm their stability. The calculated relative and free energies of the four conformers and their room temperature equilibrium populations are given in Table S1. Electronic transition and rotational strength were calculated using TDDFT at the CAM-B3LYP/def2SVP level with consideration of the methanol solvent effect using the IEFPCM [13]. Boltzmann-weighting of UV and ECD spectra were performed using SpecDis [13,14] with a half-bandwidth of 0.30 eV and UV correction of -6 nm. Specific rotations were calculated for each of conformers **1a**–**d** at the sodium D line (589.3 nm) using the B3LYP-D3(BJ)/6-311+G(2d,p) optimised geometries, with IEFPCM (MeOH), at two levels of theory: B3LYP/6-311+G(2d,p) and CAM-B3LYP/aug-cc-PVTZ. The Boltzmann-population weighted specific rotation values, +49.5 and +45.8, respectively (Table S2) were in close agreement with the value previously reported for **1** (+47.7) [2] and approximately twice the observed magnitude for **1** in the current study (+23.7).

### 3.5. Antimicrobial Testing of Extracts

Following extraction and fractionation, broth microdilution testing of the extracts was performed to ascertain the antimicrobial activity of each of the fractions against *S. aureus* ATCC 29,213 and *E. coli* ATCC 25,922. Antimicrobial activity was determined by calculating the minimum inhibitory concentration (MIC) and the minimum bactericidal concentration (MBC). Tests were performed in a clear, flat bottom 96-well plate (Thermo Fisher, NUN167008) and bacterial growth determined within each well by measuring absorbance at 600 nm using an Enspire plate reader (Perkin Elmer, MA, USA).

Extracts were obtained as dried samples which were dissolved in DMSO to make a 25 mg/mL solution and then further diluted into Mueller Hinton broth II (MHB) to achieve a maximum concentration of 2 mg/mL. Each extract (200 µL) was added to two adjacent wells along the top of the 96-well plate. MHB (100 µL) was then added to the remaining wells and extract solution (100 µL) serially diluted two-fold down the plate and discarded, leaving the last row as a growth control. Aliquots of bacteria at an optical density at 600 nm of 0.01 (approximately 1 × 10^6^ colony forming units (CFU)/mL) were then added to all the wells. This gave a maximum concentration of 1 mg/mL and a minimum concentration of 16 µg/mL. An additional plate contained a sterile control row and a negative control well containing DMSO. The maximum volume/volume concentration of DMSO in all extracts was 4%, therefore the negative control was tested at an identical concentration.

Absorbance was measured at 0, 2, 4 and 20 h, to determine MIC, between which times the plates were incubated at 37 °C with shaking at 100 rpm. Following the 20 h time point, 10 µL of liquid from all wells showing inhibition of bacterial growth was pipetted onto a plate of MH agar. The agar plates were placed inside a biosafety cabinet until all liquid had evaporated and then incubated inverted at 37 °C for 16–20 h and the MBC was measured [15].

### 3.6. Antimicrobial Assays of Pure Compounds

Antimicrobial evaluation against *S. aureus* ATCC 43,300 (MRSA), *E. coli* ATCC 25,922, *P. aeruginosa* ATCC 27,853, *Klebsiella pneumoniae* ATCC 700,603, *Acinetobacter baumannii* ATCC 19,606, *Candida albicans* ATCC 90,028 and *Cryptococcus neoformans* ATCC 208,821 was undertaken at the Community for Open Antimicrobial Drug Discovery at The University of Queensland (Queensland, Australia) according to their standard protocols [16]. For antimicrobial assays, the tested strains were cultured in either Luria broth (LB) (In Vitro Technologies, USB75852, Victoria, Australia), nutrient broth (NB) (Becton Dickson, 234,000, New South Wales, Australia), or MHB at 37 °C overnight. A sample of culture was then diluted 40-fold in fresh MHB and incubated at 37 °C for 1.5−2 h. The compounds were serially diluted 2-fold across the wells of 96-well plates (Corning 3641, nonbinding surface), with compound concentrations ranging from 0.015 to 64 μg/mL, plated in duplicate. The resultant mid log phase cultures were diluted to the final concentration of 1 × 10^6^ CFU/mL; then, 50 μL were added to each well of the compound containing plates giving a final compound concentration range of 0.008–32 μg/mL and a cell density of 5 × 10^5^ CFU/mL. All plates were then covered and incubated at 37 °C for 18 h. Resazurin was added at 0.001% final concentration to each well and incubated for 2 h before MICs were read by eye.

For the antifungal assay, fungi strains were cultured for 3 days on YPD agar at 30 °C. A yeast suspension of 1 × 10^6^ to 5 × 10^6^ CFU/mL was prepared from five colonies. These stock suspensions were diluted with yeast nitrogen base (YNB) (Becton Dickinson, 233520, New South Wales, Australia) broth to a final concentration of 2.5 × 10^3^ CFU/mL. The compounds were serially diluted 2-fold across the wells of 96-well plates (Corning 3641, nonbinding surface), with compound concentrations ranging from 0.015 to 64 μg/mL and final volumes of 50 μL, plated in duplicate. Then, 50 μL of the fungi suspension that was previously prepared in YNB broth to the final concentration of 2.5 × 10^3^ CFU/mL were added to each well of the compound-containing plates, giving a final compound concentration range of 0.008–32 μg/mL. Plates were covered and incubated at 35 °C for 36 h without shaking. *C. albicans* MICs were determined by measuring the absorbance at OD_530_. For *C. neoformans*, resazurin was added at 0.006% final concentration to each well and incubated for a further 3 h before MICs were determined by measuring the absorbance at OD_570−600_.

Colistin and vancomycin were used as positive bacterial inhibitor standards for Gram-negative and Gram-positive bacteria, respectively. Fluconazole was used as a positive fungal inhibitor standard for *C. albicans* and *C. neoformans*. The antibiotics were provided in 4 concentrations, with 2 above and 2 below its MIC value, and plated into the first 8 wells of Column 23 of the 384-well NBS plates. The quality control (QC) of the assays was determined by the antimicrobial controls and the Z’-factor (using positive and negative controls). Each plate was deemed to fulfil the quality criteria (pass QC), if the Z’-factor was above 0.4, and the antimicrobial standards showed full range of activity, with full growth inhibition at their highest concentration, and no growth inhibition at their lowest concentration.

## 4. Conclusions

Screening of the ICMP collection in pursuit of novel antimicrobials led to the isolation of a γ-lactone with a furo[3,4-*b*]pyran-5-one bicyclic ring system (**1**), two hydroxymelleins (**2** and **3**) and an isobenzofuranone (**4**) from the fungal isolate *Microcera larvarum*. Upon structure elucidation by NMR, the structure of theissenolactone A (**7**) was reassigned as **1**. The absolute configuration of **1** was then determined using the chiral derivatizing agent MPA and further supported by a combination of experimental and TDDFT calculated ECD spectroscopy.

## Figures and Tables

**Table 1:** Comparison of ^1^H-NMR data of **1** with **5** and **6** in CDCl_3_.

Position	δ_H_ (m, *J* in Hz)		
	1 ^a^	5 ^b^	6 ^b^
1	-	-	-
2	3.45 (dd, 9.0, 7.7)	3.45 (dd, 9.0, 7.3)	3.87–3.85 (m)
3	3.34 (ddd, 11.0, 9.0, 5.0)	3.30 (dddd, 11.0, 9.0, 5.0, 2.4)	3.74–3.71 (m)
4	2.64 (ddd, 13.4, 5.0, 2.0), 1.73 (ddd, 13.4, 11.0, 6.1)	2.62 (ddd, 13.3, 5.0, 2.0), 1.71 (ddd, 13.3, 11.0, 6.1)	2.62–2.57 (m), 2.03 (ddd, 15.3, 8.0, 3.6)
5	2.90 (ddd, 6.1, 4.1, 2.0)	2.87 (ddd, 6.1, 3.1, 2.0)	2.62–2.57 (m)
6	-	-	-
7	4.58 (q, 7.0)	4.50 (dq, 3.1, 6.7)	4.52 (dq, 3.6, 6.7)
8	4.04 (d, 4.1)	4.12 (dd, 3.1, 3.1)	4.21 (dd, 3.6, 3.6)
9	1.33 (d, 7.0)	1.44 (d, 6.7)	1.52 (d, 6.7)
10	5.42 (ddq, 15.5, 7.7, 1.6)	5.44 (ddq, 15.3, 7.3, 1.2)	5.52 (ddq, 15.3, 4.9, 1.8)
11	5.89 (dq, 15.5, 6.6)	5.85 (dq, 15.3, 6.1)	5.84 (dq, 15.3, 6.7)
12	1.77 (dd, 6.6, 1.6)	1.76 (br d, 6.1)	1.75 (dd, 6.7, 1.8)
OH	1.75–1.70 (m)	1.96 (d, 2.4)	1.72 (d, 4.3)

^a^ Data recorded at 500 MHz in CDCl_3_. ^b^ Data from Gao et al. [1] recorded at 400 MHz in CDCl_3_.

**Table 2:** Comparison of ^13^C-NMR data of **1** with **5** and **6** in CDCl_3_.

Position	1 ^a^	5 ^b^	6 ^b^
1	-	-	-
2	81.5	78.2	76.9
3	66.6	66.5	65.2
4	28.3	28.4	27.8
5	39.0	42.9	38.2
6	176.0	176.3	177.0
7	80.5	80.9	78.5
8	77.7	74.6	75.1
9	17.6	13.5	13.4
10	127.8	127.8	127.7
11	133.0	131.4	128.7
12	18.2	18.0	18.0

^a^ Data recorded at 125 MHz in CDCl_3_. ^b^ Data from Gao et al. [1] recorded at 100 MHz in CDCl_3_.

**Table 3:** Comparison of ^1^H and ^13^C-NMR data (CD_3_OD) of **1** with theissenolactone A.

Position	1		Theissenolactone A (7)	
	δ_H_ (m, *J* in Hz) ^a^	δ_C_^b^	δ_H_ (m, *J* in Hz) ^c^	δ_C_^d^
1	-	-	-	-
2	3.46 (dd, 9.0, 7.6)	81.9	3.44 (dd, 7.9, 7.7)	81.9
3	3.15 (ddd, 11.0, 9.0, 5.0)	68.0	3.11–3.15 (m)	68.0
4	2.45 (ddd, 13.4, 5.0, 2.0), 1.75 (ddd, 13.4, 11.0, 6.5)	30.2	2.43 (dd, 11.2, 5.0), 1.74 (d, 11.2)	30.1
5	3.13 (ddd, 6.5, 4.3, 2.0)	40.4	3.08–3.12 (m)	40.4
6	-	178.8	-	178.9
7	4.53 (q, 6.8)	82.2	4.50 (q, 6.8)	82.3
8	4.10 (d, 4.3)	78.9	4.07 (s)	78.9
9	1.34 (d, 6.8)	17.4	1.31 (d, 6.8)	17.4
10	5.48 (ddd, 15.3, 7.6, 1.6)	129.8	5.45 (dd, 15.8, 7.9)	129.8
11	5.80 (dq, 15.3, 6.5)	130.7	5.76 (dq, 15.8, 6.7)	130.7
12	1.73 (br d, 6.5)	18.1	1.70 (d, 6.7)	18.1

^a^ Data recorded at 500 MHz in CD_3_OD. ^b^ Data recorded at 125 MHz in CD_3_OD. ^c^ Data from Liang et al. [2] recorded at 500 MHz in CD_3_OD. ^d^ Data from Liang et al. [2] recorded at 125 MHz in CD_3_OD.

**Table 4:** Antimicrobial activity of compounds **1**–**3**.

Compound	MIC (µg/mL)						
	*S. a ^a^*	*P. a ^b^*	*E. c ^c^*	*K. p ^d^*	*A. b ^e^*	*C. a ^f^*	*C. n ^g^*
**1**	>32 ^h^	>32 ^h^	>32 ^h^	>32 ^h^	>32 ^h^	>32 ^h^	>32 ^h^
**2**	>32 ^h^	>32 ^h^	>32 ^h^	>32 ^h^	>32 ^h^	>32 ^h^	>32 ^h^
**3**	>32 ^h^	>32 ^h^	>32 ^h^	>32 ^h^	>32 ^h^	>32 ^h^	>32 ^h^

Key: All values presented as the mean (*n* = 2). ^a^ *Staphylococcus aureus* ATCC 43,300 (MRSA); ^b^ *Pseudomonas aeruginosa* ATCC 27853; ^c^ *Escherichia coli* ATCC 25922; ^d^ *Klebsiella pneumoniae* ATCC 700,603; ^e^ *Acinetobacter baumanii* ATCC 19,606; ^f^ *Candida albicans* ATCC 90028; ^g^ *Cryptococcus neoformans* ATCC 208821; ^h^ not active at a single dose test of 32 µg/mL.

## Supplementary Materials

The following are available online. The ^1^H, ^13^C, COSY, HSQC, HMBC and NOESY NMR spectra and HRESIMS spectrum for compound **1** are available along with the calculated relative free energies and specific rotations for the four conformers of **1**.
