# Supplementary material for: A Revised Structure and Assigned Absolute Configuration of Theissenolactone A
Source: Molecules. 2020 Oct 20;25(20):4823. doi: 10.3390/molecules25204823 (PMC7587954; doi:10.3390/molecules25204823)
Supplement: Supplementary file 1 [file molecules-25-04823-s001.pdf]

Supporting Information

A revised structure and assigned absolute configuration of theissenolactone A

Melissa M. Cadelis *[1,2], Soeren Geese [2], Lauren Gris [3], Bevan S. Weir [4], Brent R. Copp †[1] and Siouxsie Wiles *†[2]

[1] School of Chemical Sciences, University of Auckland, Private Bag 92019, Auckland 1142, Aotearoa New Zealand; m.cadelis@auckland.ac.nz; b.copp@auckland.ac.nz

[2] Bioluminescent Superbugs Lab, School of Medical Sciences, University of Auckland, Private Bag, 92019, Auckland 1142, Aotearoa New Zealand; s.geese@auckland.ac.nz; s.wiles@auckland.ac.nz

[3] Department of Fine and Industrial Organic Chemistry, SIGMA Clermont, Campus des Cézeaux, CS 20265, 63178 Aubière, France; lauren.gris85@laposte.net

[4] Manaaki Whenua/Landcare Research Ltd., Private Bag 92170, Auckland 1142, Aotearoa New Zealand; WeirB@landcareresearch.co.nz

* Correspondence: m.cadelis@auckland.ac.nz and s.wiles@auckland.ac.nz

† These authors contributed equally to this work.

Contents

Table S1 Calculated relative and free energies of the four conformers and their room temperature equilibrium populations. **S2**

Table S2 Calculated specific rotations for each of conformers **1a–d** at two levels of theory: B3LYP/6-311+G(2d,p) and CAM-B3LYP/aug-cc-PVTZ **S2**

Figure S1 Structures of conformers **1a–d** **S3**

Figure S2 ^{1}H NMR spectrum (500 MHz, CD_3OD) of **1** **S4**

Figure S3 ^{13}C NMR spectrum (125 MHz, CD_3OD) of **1** **S5**

Figure S4 ^{1}H NMR spectrum (500 MHz, $CDCl_3$) of **1** **S6**

Figure S5 ^{13}C NMR spectrum (125 MHz, $CDCl_3$) of **1** **S7**

Figure S6 COSY spectrum ($CDCl_3$) of **1** **S8**

Figure S7 HSQC spectrum ($CDCl_3$) of **1** **S9**

Figure S8 HMBC spectrum ($CDCl_3$) of **1** **S10**

Figure S9 NOESY spectrum ($CDCl_3$) of **1** **S11**

Figure S10 HRESIMS of **1** **S12**

Table S1 Calculated relative and free energies of the four conformers **1a–d** and their room temperature equilibrium populations.

Conformer	E [a] (Hartree)	C [b] (Hartree)	G [c]	ΔG (kJ/mol) [d]	%P [e]
**1a**	-729.9911056	0.21852	-729.7725856	0.0000	86.33
**1b**	-729.9877297	0.218639	-729.7699137	7.0151	5.10
**1c**	-729.9884346	0.217816	-729.7697956	7.3253	4.50
**1d**	-729.9876276	0.217923	-729.7697046	7.5640	4.08

[a] Electronic energy obtained at B3LYP-D3 6-311+G(2d,p) IEFPCM (MeOH); [b] Thermal correction to Gibbs free energy at same level of theory; [c] Gibbs free energy; [d] The relative Gibbs free energy; [e] Boltzmann population at 298.15K.

Table S2 Calculated specific rotations for each of conformers **1a–d** at two levels of theory: B3LYP/6-311+G(2d,p) and CAM-B3LYP/aug-cc-PVTZ

Conformer	CAM-B3LYP[a]	B3LYP[b]
**1a**	49.33	51.84
**1b**	39.22	50.36
**1c**	-7.84	5.31
**1d**	37.31	46.40
average[c]	45.8	49.5

[a] CAM-B3LYP/aug-cc-PVTZ with IEFPCM (MeOH), [b] B3LYP/6-311+G(2d,p) with IEFPCM (MeOH), [c] Boltzmann population average (298.15K)

1a

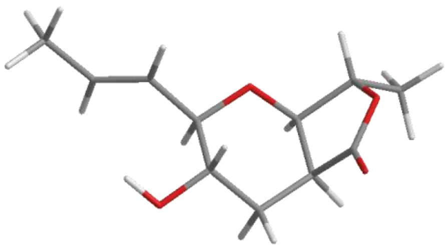

1c

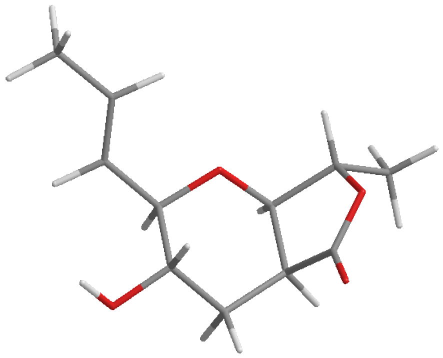

1b

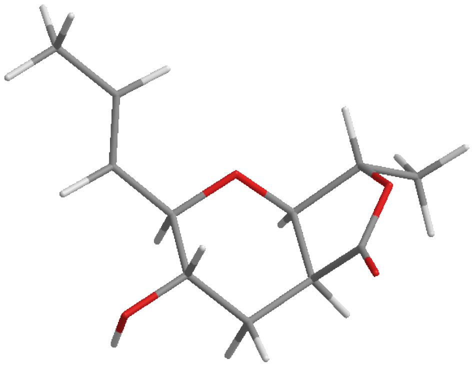

1d

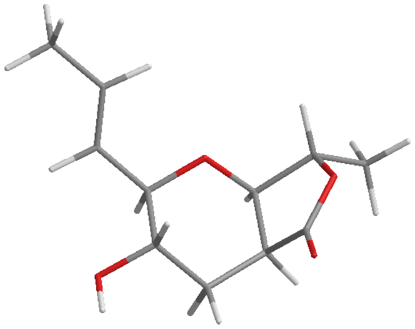

Figure S1 Structures of conformers **1a–d**

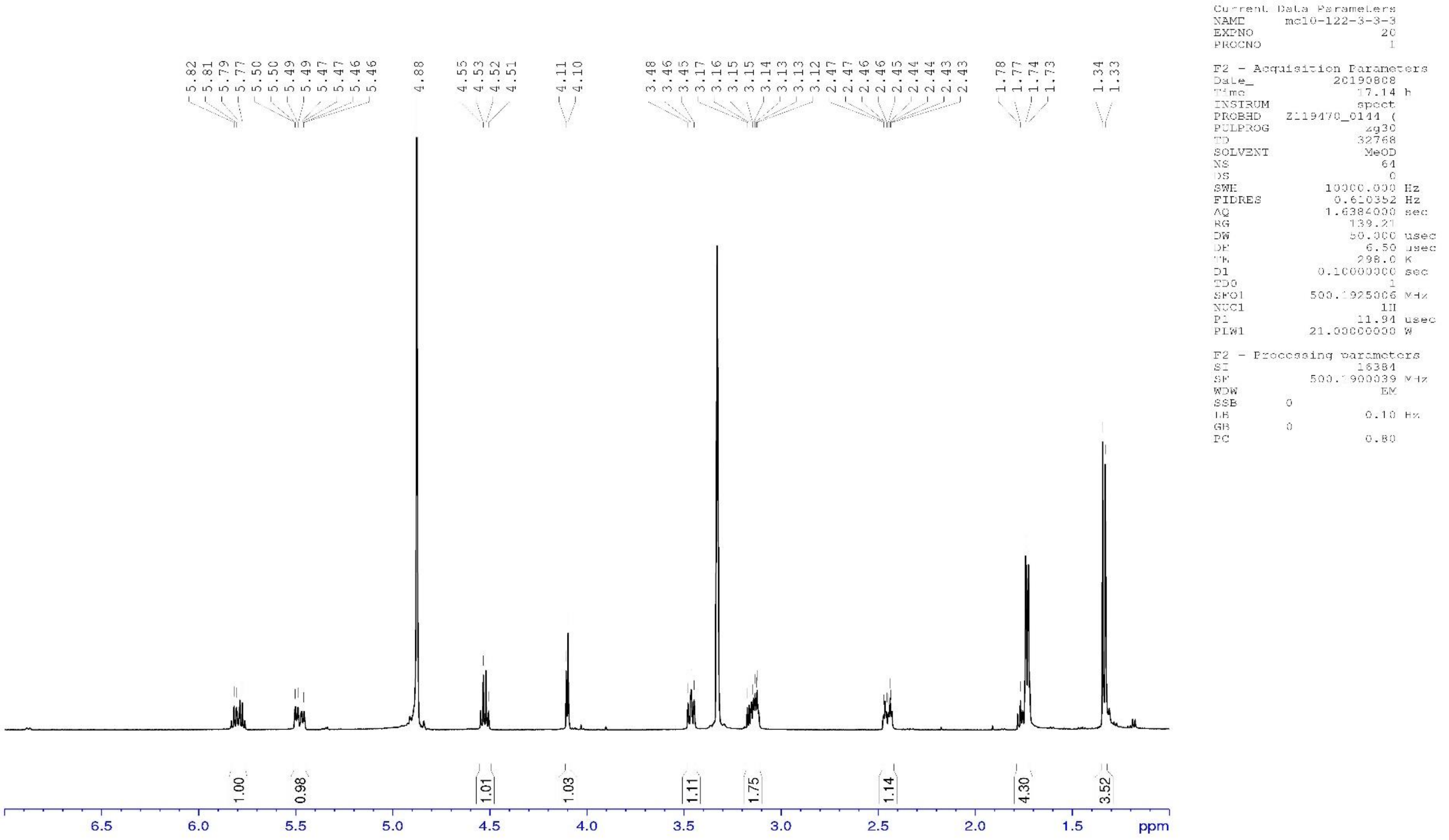

Figure S2 ^{1}H NMR spectrum (500 MHz, CD_3OD) of **1**

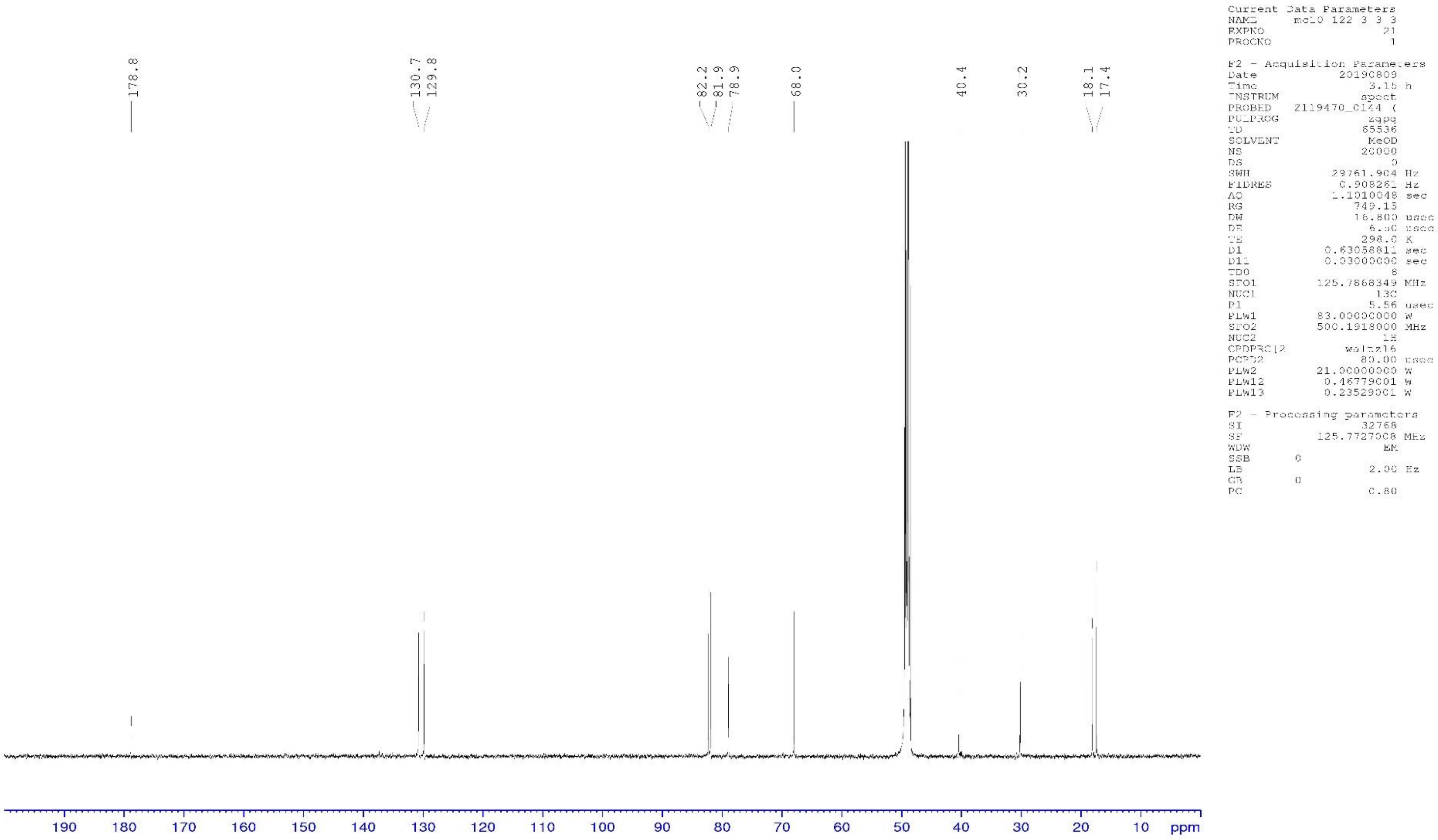

Figure S3 ^{13}C NMR spectrum (125 MHz, CD_3OD) of **1**

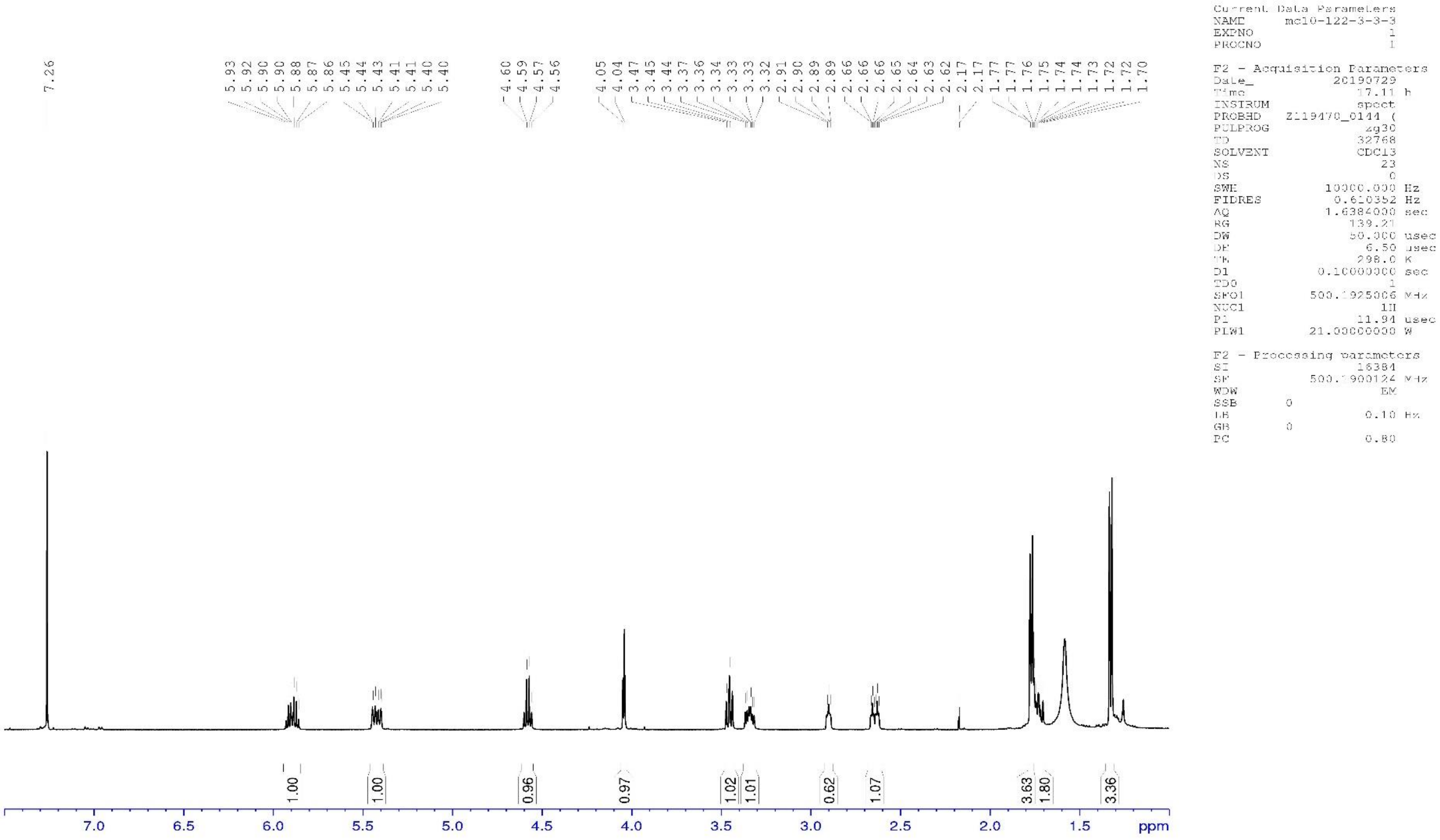

Figure S4 ^{1}H NMR spectrum (500 MHz, $CDCl_3$) of **1**

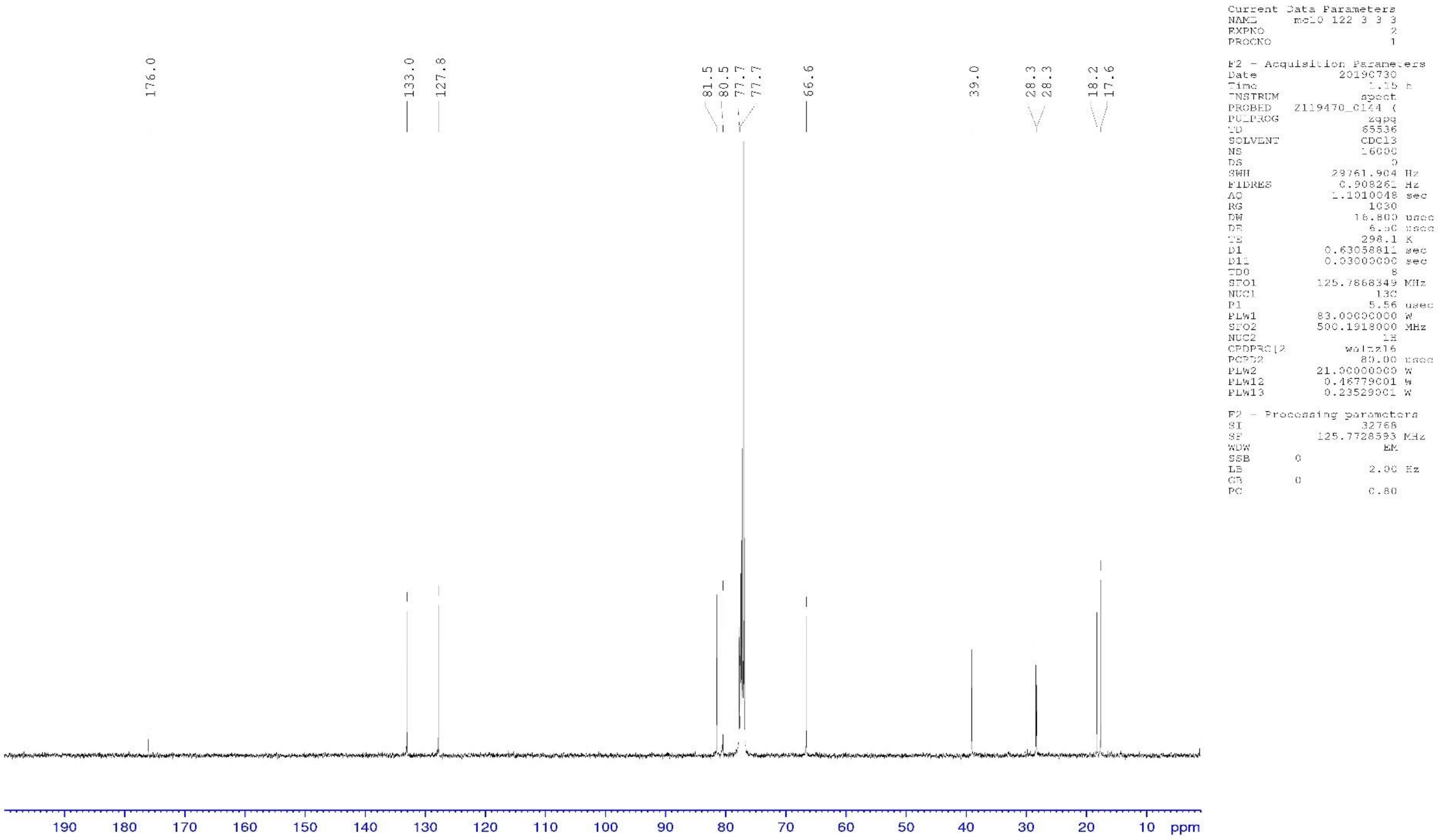

Figure S5 ^{13}C NMR spectrum (125 MHz, $CDCl_3$) of **1**

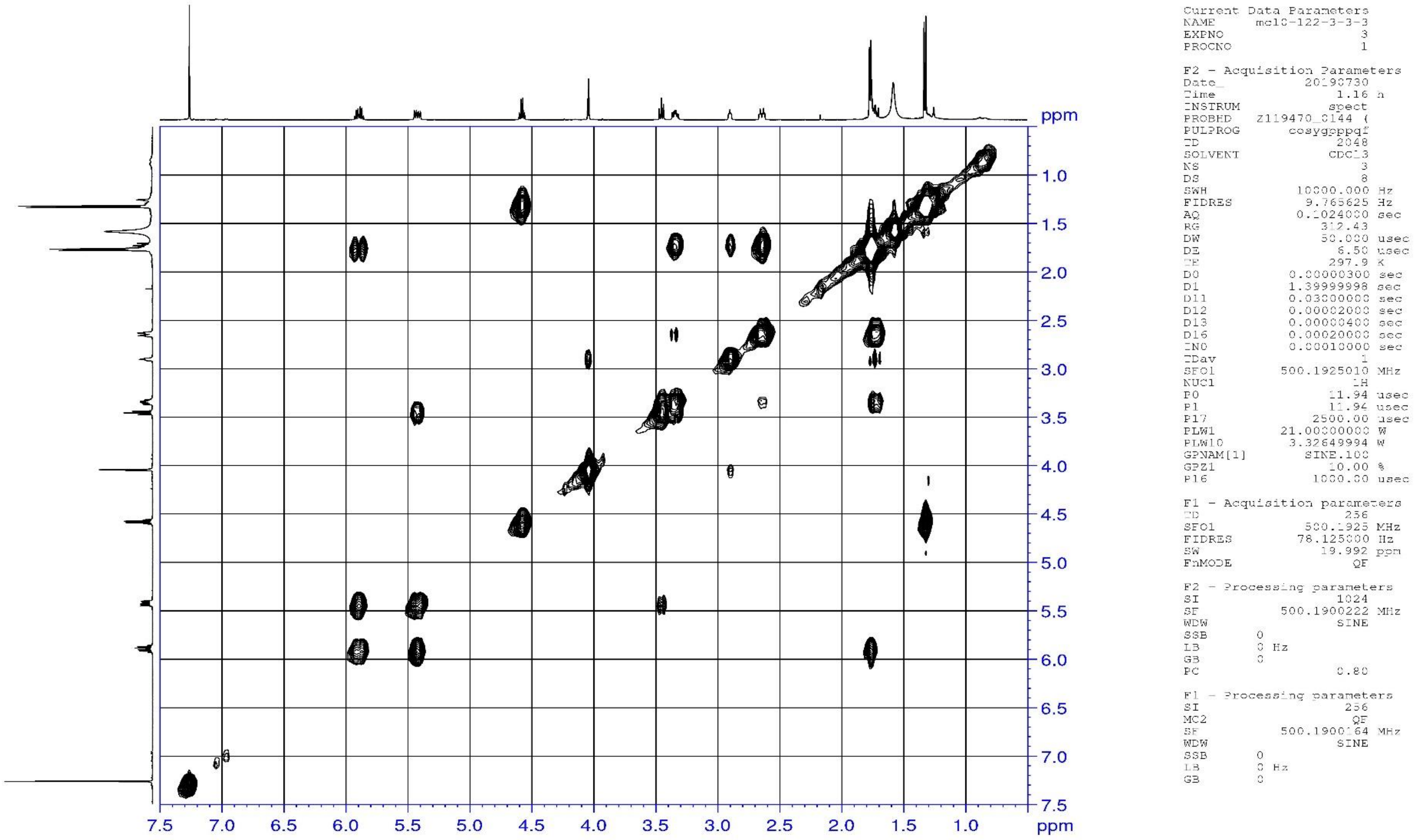

Figure S6 COSY spectrum ($CDCl_3$) of **1**

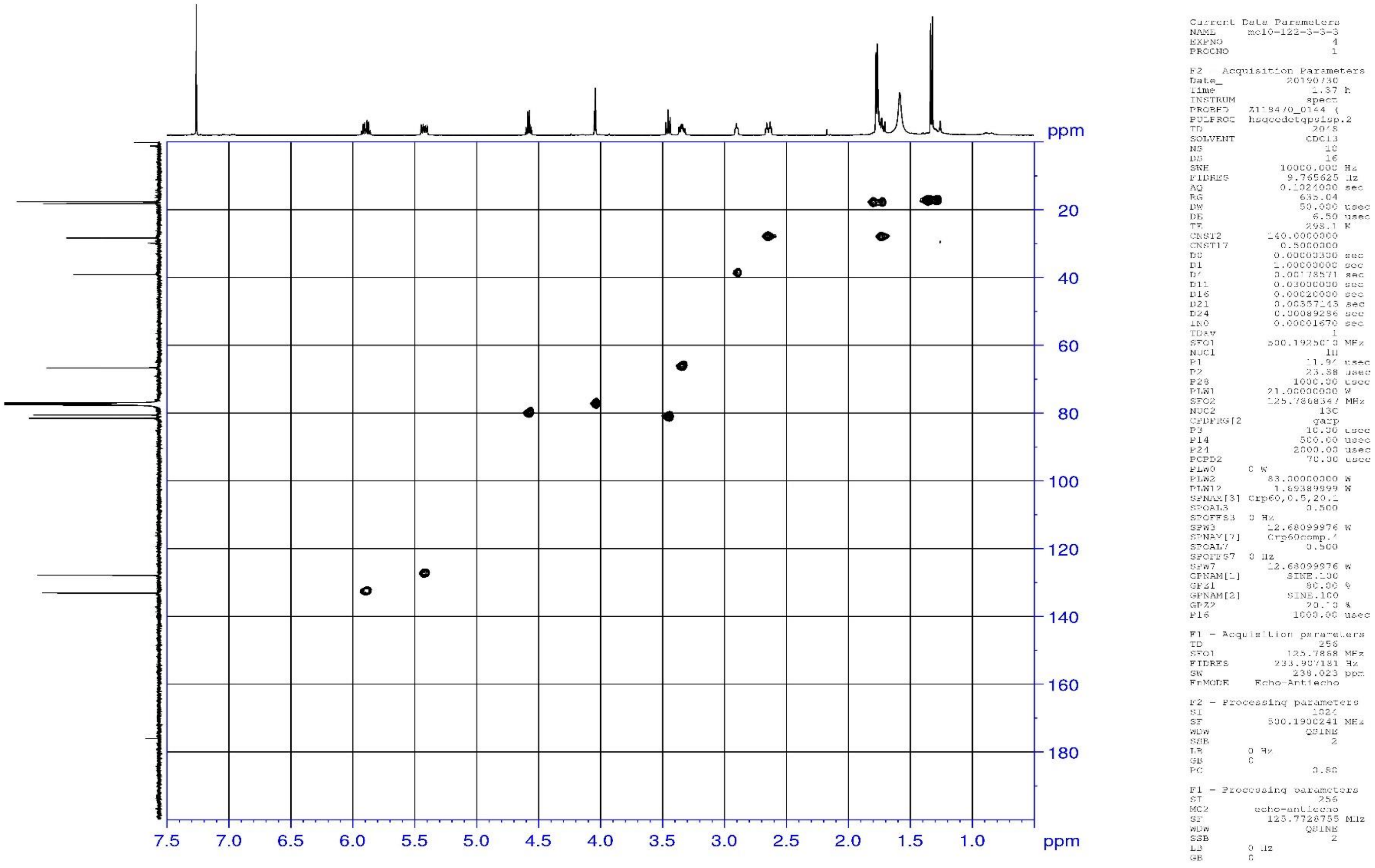

Figure S7 HSQC spectrum ($CDCl_3$) of **1**

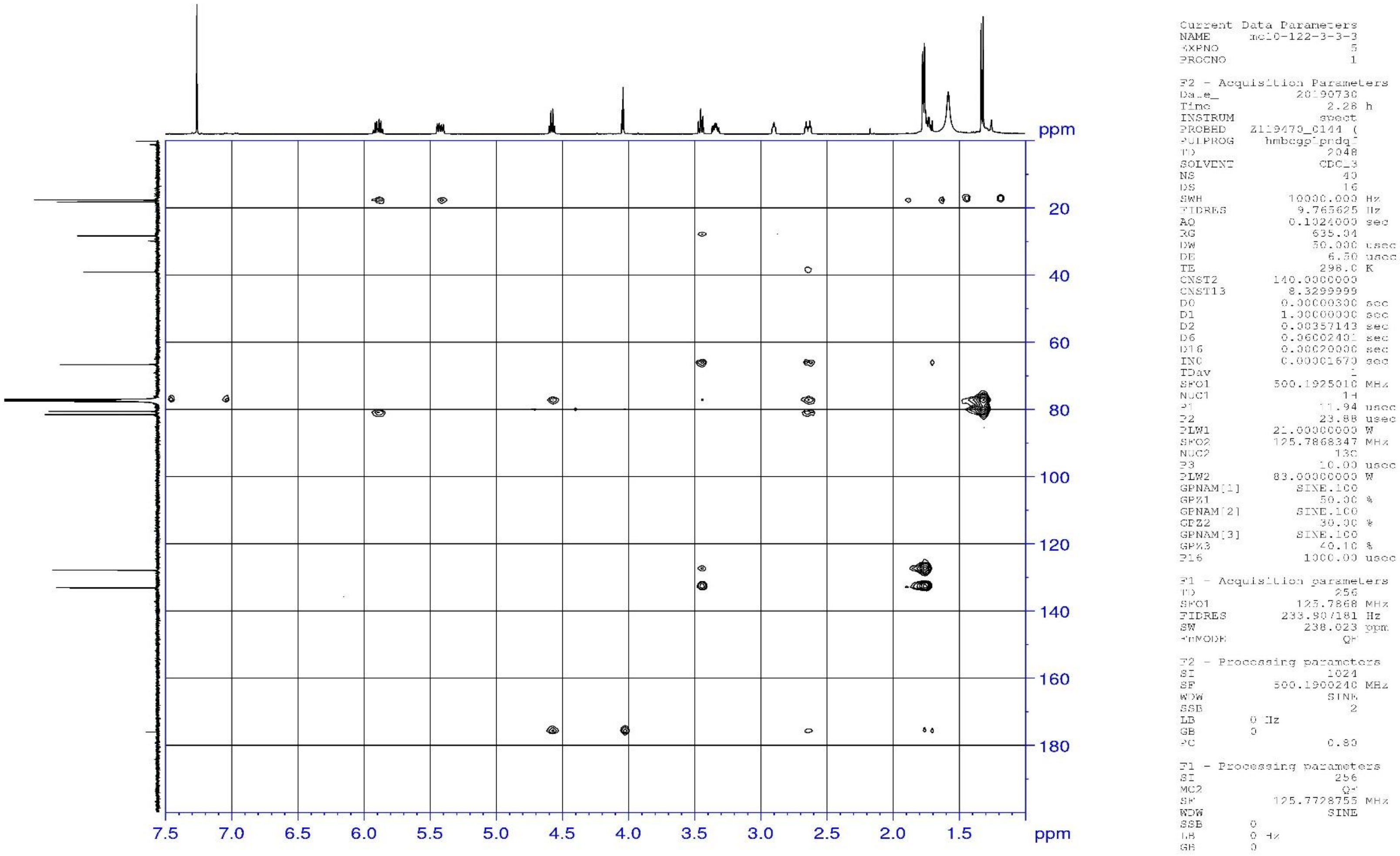

Figure S8 HMBC spectrum ($CDCl_3$) of **1**

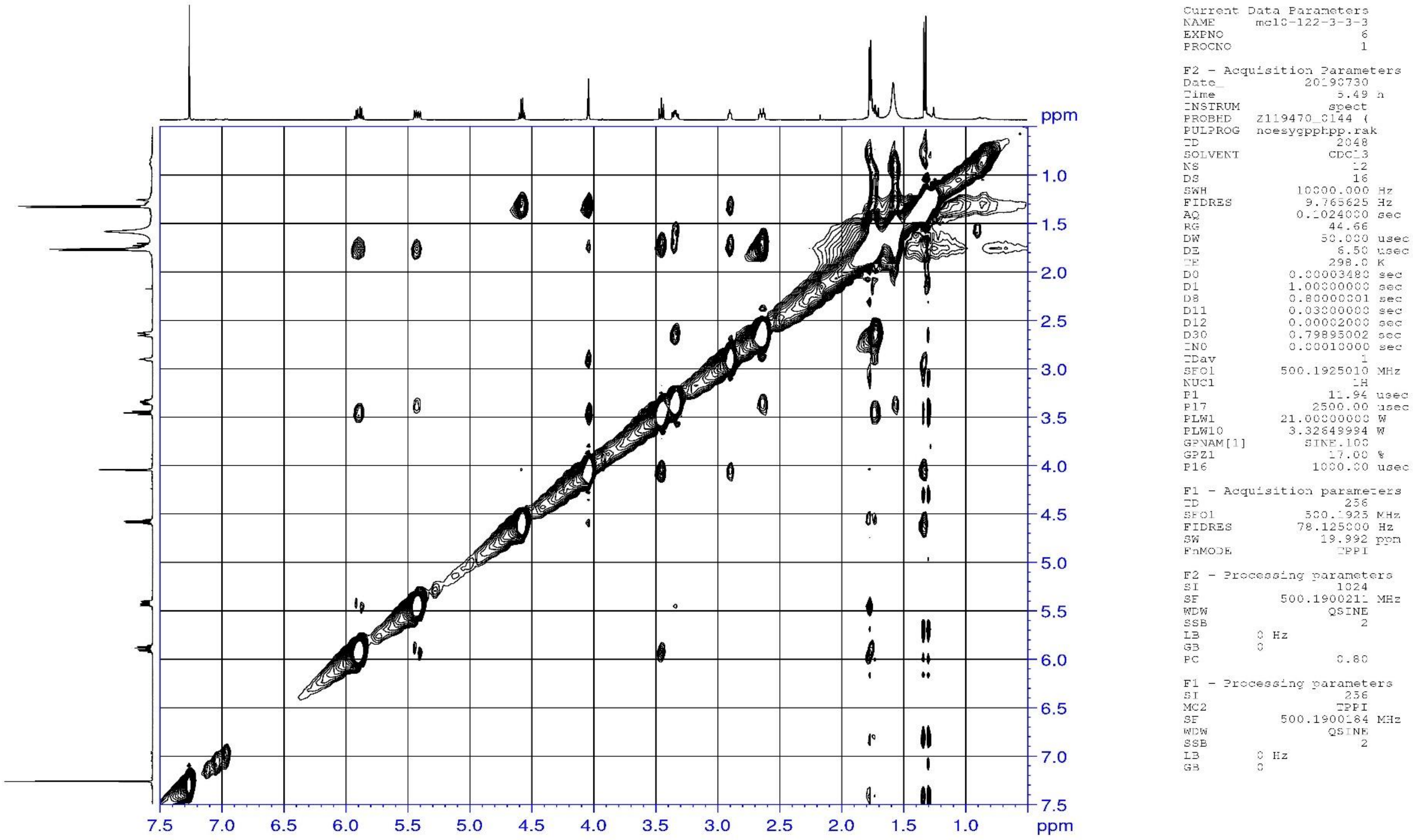

Figure S9 NOESY spectrum ($CDCl_3$) of **1**

Acquisition Parameter					
Source Type	ESI	Ion Polarity	Positive	Set Nebulizer	0.4 Bar
Focus	Not active	Set Capillary	4500 V	Set Dry Heater	180 °C
Scan Begin	50 m/z	Set End Plate Offset	-500 V	Set Dry Gas	4.0 l/min
Scan End	1000 m/z	Set Collision Cell RF	150.0 Vpp	Set Divert Valve	Waste

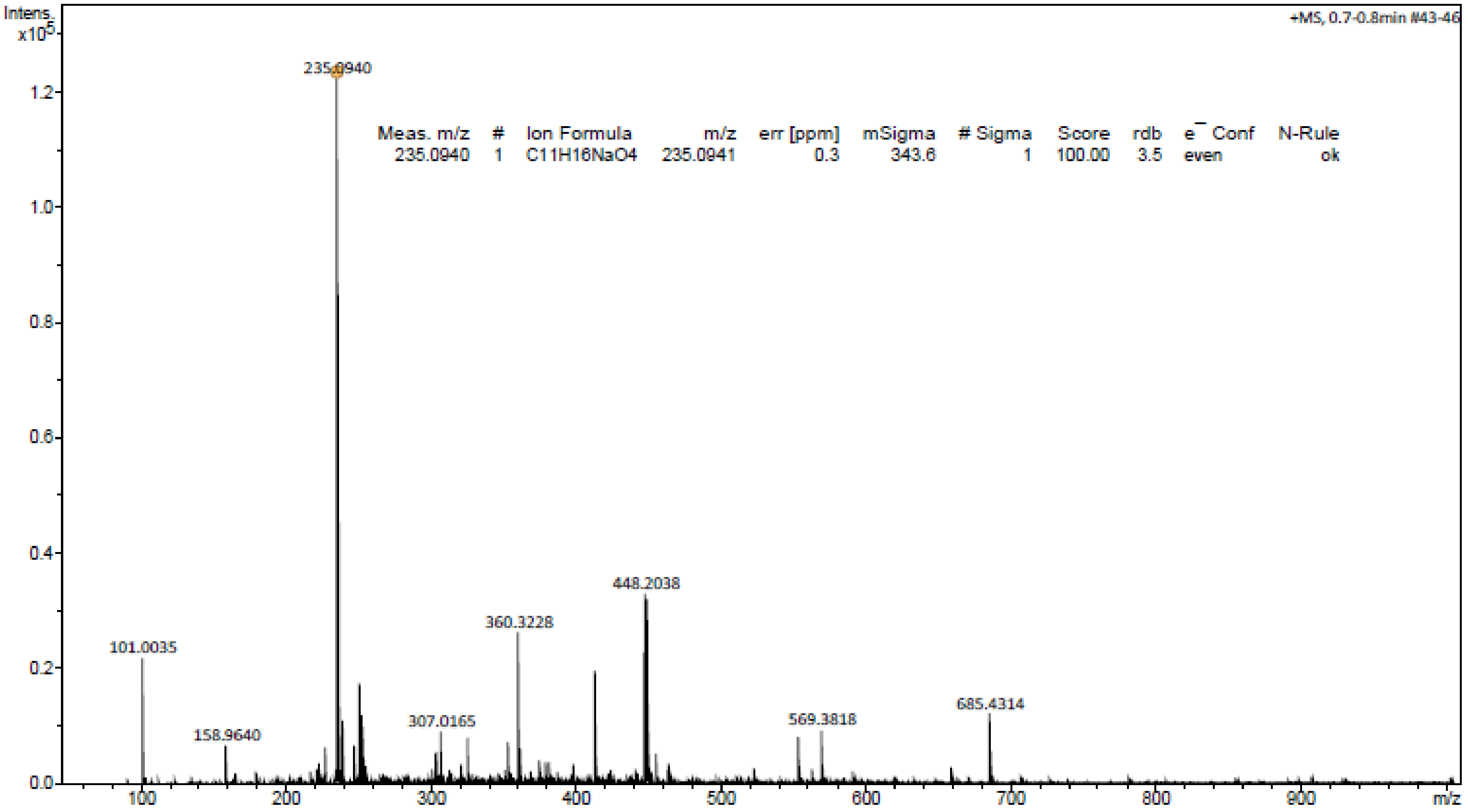

Figure S10 HRESIMS of **1**
